# Safety and efficacy of exercise training in adults with Pompe disease: evalution of endurance, muscle strength and core stability before and after a 12 week training program

**DOI:** 10.1186/s13023-015-0303-0

**Published:** 2015-07-19

**Authors:** Linda E. M. van den Berg, Marein M. Favejee, Stephan C. A. Wens, Michelle E. Kruijshaar, Stephan F. E. Praet, Arnold J. J. Reuser, Johannes B. J. Bussmann, Pieter A. van Doorn, Ans T. van der Ploeg

**Affiliations:** Center for Lysosomal and Metabolic Diseases, Department of Pediatrics, Erasmus MC University Medical Center – Sophia Children’s Hospital, PO Box 2040, 3000 CA Rotterdam, The Netherlands; Department of Rehabilitation Medicine & Physical Therapy, Erasmus MC University Medical Center, PO Box 2040, 3000 CA Rotterdam, The Netherlands; Center for Lysosomal and Metabolic Diseases, Department of Neurology, Erasmus MC University Medical Center, PO Box 2040, 3000 CA Rotterdam, The Netherlands; Center for Lysosomal and Metabolic Diseases, Department of Clinical Genetics, Erasmus MC University Medical Center, PO Box 2040, 3000 CA Rotterdam, The Netherlands

**Keywords:** Glycogen storage disorder type II, Endurance training, Strength training, Core stability, Muscle function

## Abstract

**Background:**

Pompe disease is a proximal myopathy. We investigated whether exercise training is a safe and useful adjuvant therapy for adult Pompe patients, receiving enzyme replacement therapy.

**Methods:**

Training comprised 36 sessions of standardized aerobic, resistance and core stability exercises over 12 weeks. Before and after, the primary outcome measures safety, endurance (aerobic exercise capacity and distance walked on the 6 min walk test) and muscle strength, and secondary outcome measures core stability, muscle function and body composition, were evaluated.

**Results:**

Of 25 patients enrolled, 23 successfully completed the training. Improvements in endurance were shown by increases in maximum workload capacity (110 W before to 122 W after training, [95 % CI of the difference 6 · 0 to 19 · 7]), maximal oxygen uptake capacity (69 · 4 % and 75 · 9 % of normal, [2 · 5 to 10 · 4]), and maximum walking distance (6 min walk test: 492 meters and 508, [−4 · 4 to 27 · 7] ). There were increases in muscle strength of the hip flexors (156 · 4 N to 180 · 7 N [1 · 6 to 13 · 6) and shoulder abductors (143 · 1 N to 150 · 7 N [13 · 2 to 35 · 2]). As an important finding in secondary outcome measures the number of patients who were able to perform the core stability exercises rose, as did the core stability balancing time (*p* < 0.05, for all four exercises). Functional tests showed small reductions in the time needed to climb four steps (2 · 4 sec to 2 · 1, [− 0 · 54 to −0 · 04 ]) and rise to standing position (5 · 8 sec to 4 · 8, [−2 · 0 to 0 · 0]), while time to run, the quick motor function test results and body composition remained unchanged.

**Conclusions:**

Our study shows that a combination of aerobic, strength and core stability exercises is feasible, safe and beneficial to adults with Pompe disease.

## Background

Pompe disease (glycogen storage disease type II, acid maltase deficiency) (OMIM # 232300) is a rare metabolic myopathy caused by glycogen accumulation resulting from deficiency of lysosomal acid α-glucosidase (GAA). It presents as a wide clinical spectrum, the most prominent symptoms in adults being proximal skeletal muscle weakness and respiratory problems [1, 2]. Skeletal muscle weakness typically fits a pattern of limb-girdle myopathy, with the abdominal and paraspinal muscles and the musculature of the hip being the most affected muscle groups [3–5].

Enzyme replacement therapy (ERT) with recombinant human acid α-glucosidase (Myozyme/Lumizyme) was approved for the treatment of Pompe disease in 2006. In adults, ERT has been shown to elicit positive effects on skeletal muscle strength, walking distance, respiratory function and survival [6–8]. Patients’ fitness and physical functioning may be further supported by treatments additional to ERT, such as exercise training. Although some recent studies suggest that exercise training may be beneficial, evidence is still limited [9, 10].

A recent study on common clinical practice in the Netherlands showed that there is a lack of uniformity in the type of physical therapy training programs applied, and that physical therapists and patients all seek guidance and standardization [11]. We therefore aimed to determine whether a standardized and well-structured exercise intervention program combining aerobic, resistance and core stability exercises was feasible and safe, and whether it added value to treatment with ERT alone. In a group of relatively mildly affected adult Pompe patients receiving ERT for more than a year, we evaluated the effects of such a regime on endurance, muscle strength and function, core stability, and body composition.

## Methods

### Patients

Patients were recruited at the Centre for Lysosomal and Metabolic Diseases, Erasmus MC University Medical Centre, Rotterdam, the Dutch national referral centre for patients with Pompe disease.

There were three inclusion criteria:

1. A confirmed diagnosis of Pompe disease measured by decreased acid α-glucosidase activity in leukocytes or fibroblasts, and mutation analysis;
2. Age > 17 years;
3. Treatment with ERT for at least 52 weeks

There were four exclusion criteria:

1. The use of walking-aids or a wheelchair;
2. Ventilator-dependency;
3. Concurrent medical conditions;
4. Participation in other exercise-training programs.

The study was approved by the Ethical Committee at Erasmus MC University Medical Centre. Informed consent was obtained from all patients.

### Study design and intervention

**Fig. 1:**
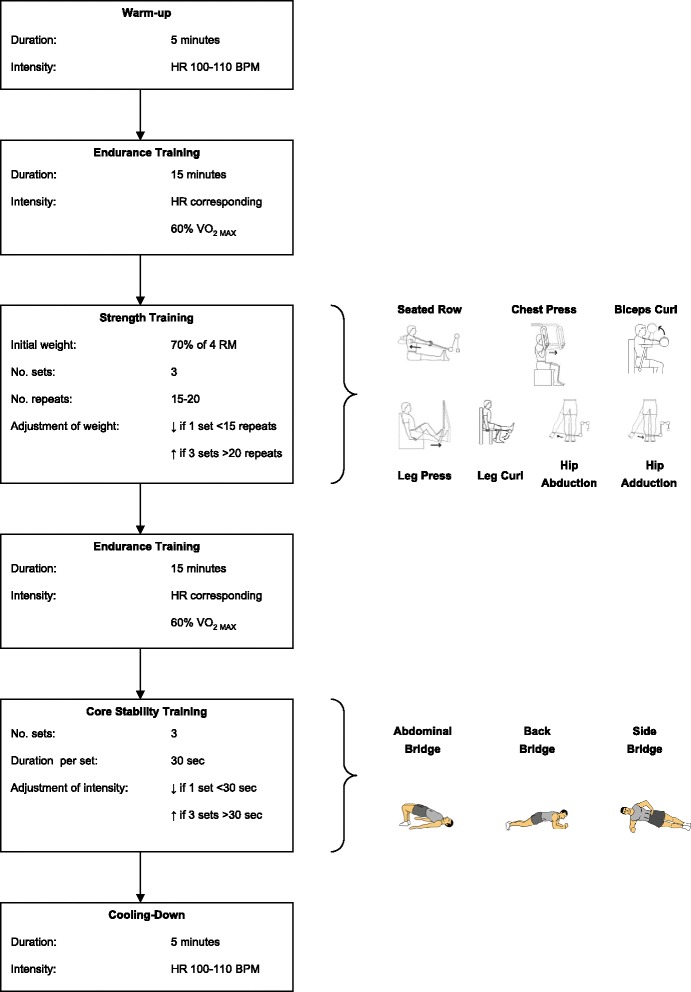
Flowchart for the standardized exercise-training regime combining aerobic, resistance and core stability exercises

Three times a week for 12 weeks, all patients followed a standardized training program that was provided under the supervision of physical therapists at carefully selected sports or fitness centres near the patients’ homes. To ensure the uniformity of the program and its supervision, all therapists attended a one-day instruction program at Erasmus MC University Medical Centre. The training program is depicted in Fig. 1. The first training session was on-site supervised by one of the researchers from Erasmus MC (LvdB, MF), who subsequently attended each training site every two weeks to monitor proper conduct of the program.

Patients were randomly subdivided into two groups: group 1 (*n* = 13), which started the training program at week 1; and group 2 (*n* = 12), which started at week 13. The staggered start of training allowed us to investigate whether any improvement observed in the training period could also be attributed to ERT. Furthermore, the duration of the effect of the training program can be evaluated from the follow-up of patients in group 1 after they stop training in week 12.

To assess the effects of the program, each patient visited our centre (Erasmus MC) on two separate days in weeks 0, 12 and 24. The primary endpoints of this study were safety, endurance and muscle strength. Secondary endpoints were core stability, muscle function, and body composition.

Under the supervision of the physical therapist, training diaries were kept by all patients, who recorded the days on which they trained, the weight and number of repeats of resistance exercises, and the perceived level of exertion. To evaluate training progress and patients’ motivation, patients were telephoned weekly.

### Assessments

#### Safety

Plasma CK was measured every two weeks as a safety marker for exercise-induced muscle damage [12], and patients were contacted every week to record potential side effects such as pain and fatigue.

#### Endurance

Aerobic (endurance) exercise capacity was determined using an incremental cycle ergometer. After 4 min of unloaded cycling on the cycloergometer (Jaeger ER 800; Erich Jaeger, Würzburg Germany) exercise intensity was increased progressively until exhaustion (i.e. ramp protocol), during continuous measurement of patients’ heart rates and ventilator parameters using spiroergometry equipment (Oxycon Pro, Jaeger, Würzburg, Germany). The rate of increase was determined considering the patient’s functional capacities and ranged from 5–20 Watts/minute. The duration of every individual test exceeded 6 min but did not take longer than 12 min. At exhaustion, the rating of exertional symptoms was assessed using the Borg scale (scale 6–20) [13], patients consistently scoring 14 or above. Maximum workload capacity (W_MAX_) and, peak oxygen uptake capacity (VO_2 PEAK_) were measured. The ventilatory threshold (VT) was assessed independently by two clinical exercise physiologists using the ventilatory equivalents method [14]. The test was considered to be maximal when one of the following criteria was met [14]:

1. heart rate > 90 % of that predicted,
2. respiratory exchange ratio (RER) > 1 · 11, or
3. VO_2_ stabilized despite increased workload.

Walking distance on the 6-min walk test (6MWT) was evaluated according to the American Thoracic Society guidelines.

#### Core Stability

To assess the dynamic balance, which reflects core stability, one physical therapist (MF) measured time in balance (in seconds) for each of the four core stability exercises of the training program (Fig. 1) [15].

#### Muscle strength

Muscle strength was assessed by one investigator (SW) using Hand-held Dynamometry (HHD). Assessments were performed in a standardized manner, and sum scores were calculated as described previously [6].

#### Muscle Function

Functional activity assessments comprised three timed tests: 10 meter running, climbing four steps, and rising from supine to standing positions [16], and the Quick Motor Function Test (QMFT), a test specifically designed and validated for Pompe patients [17]. The QMFT consists of 16 specific motor skills related to daily activities. A total score is achieved by summing the scores for each item (ranging from 0 “cannot perform” to 4 “can perform with no effort”), and is expressed as a percentage of the maximum score.

#### Body composition

Bone-mineral density (BMD) and body-composition measurements were performed conform DXA technology using a Lunar DPX densitometer and analyzed with Encore 2002 software (GE Lunar DPX, GE Health Care). Bone densitometry was performed in a standardized manner as described previously [18]. Body composition was described in terms of the mineral, lean and fat body mass (kilograms). The percentage of fat mass and, more specifically, android and gynoid fat, were calculated.

### Statistical analysis

Patient characteristics were summarized using descriptive statistics. Data for the two groups were combined after verifying that there were no significant differences between outcome measures before the start of the training (group 1 – week 0; group 2 – week 12; student’s t-test for normally distributed, and Mann–Whitney for not-normally distributed data).

Mean differences before and after the training were compared using the paired t-test for normally distributed data, and otherwise the Wilcoxon signed rank test for paired samples. For group 2, we also used these tests to compare the outcome measures before and after 12 weeks of ERT only (week 0 to 12).

Significance level was set at *p <* 0 · 05. Statistical analyses were performed using SPSS for Windows (release 17 · 0; SPSS, Inc., Chicago, IL).

## Results

### Patients

A total of 25 patients fulfilled the inclusion criteria and chose to participate in this study. Two patients did not complete the training program because they were insufficiently motivated. This left 23 patients, who successfully completed the study. Their ages ranged from 20 to 71 years (median of 46 years). They had been receiving ERT for 1 to 6 years with a median of 3 years (Table 1).

**Table 1:** Patient characteristics

	Group 1 (*n* = 12)	Group 2 (*n* = 11)	Total group (*n* = 23)	P-value*
Male gender (%)	7 (58 %)	5 (45 %)	12 (52 %)	0.54
Age in years (range)	45.4 (19.6-70.5)	46.6 (32.9-66.1)	46.0 (19.6-70.5)	0.85
Disease duration in years (range)	15.5 (8.1-28.1)	16.1 (6.0-32.1)	15.8 (6.0-32.1)	0.83
ERT duration in years (range)	3.3 (1.4-6.5)	3.0 (1.3-3.6)	3.1 (1.3-6.5)	0.96
Training sessions (max. 36)	33 (27–36)	32 (24–35)	32 (24–36)	0.70

Group 1 trained in weeks 1–12 and Group 2 trained in weeks 13–24

*ERT* enzyme replacement therapy

^*^For the difference between group 1 and 2 (chi-2 test for proportions and Wilcoxon signed rank test for continuous data)

### Effect of ERT only

During the 12 weeks before training started, patients in group 2 (ERT only) underwent the same set of assessments as in the 12 weeks of training (ERT plus training). This enabled us to use group 2 to compare the effects of ERT only with the combined effects of ERT and training. During the first 12 weeks of ERT only, we detected no significant improvements in the main outcome measures.

### Effect of training

Patients in the two randomly assigned groups were comparable in terms of age, gender, disease duration, time on ERT, number of training sessions completed, and baseline test results. This allowed us to analyse the effect of training in the total group of 23 patients.

#### Safety

During the first week of training, two patients had a high plasma CK level (10125 U/l and 6149 U/l), and also experienced muscle pain and fatigue. Over the following week, their CK-values dropped to their normal range, and the fatigue and pain disappeared. Both patients continued training. None of the other patients had pain, fatigue, or increases in plasma CK levels during the study period.

#### Endurance

All patients were able to complete the incremental cycle test without adverse events. One was excluded from the analysis because he did not reach the required maximum intensity defined in the method section. After 12 weeks of training W_MAX_, VO_2peak_ and VT improved significantly (Table 2). VO_2peak_ and VT increased both in absolute values and adjusted for body weight or as a percentage of normal values. The ratio VT/VO_2peak_ did not change, as both the numerator and the denominator increased. There were no significant differences between patients’ maximum heart rates before and after 12 weeks of training, indicating that the results were truly based on an increase in fitness rather than on greater exertions by the patients towards the end of the training period. FVC did not change. Average walking distance on the 6MWT increased by 16 meters ([4.4-27.7], p = 0.01).

**Table 2:** Aerobic fitness measured in an incremental cycle test and a 6-min walk test before and after 12 weeks of training

	Before training	After training	*P*-value**
	Mean ± SD	Mean ± SD	
Incremental cycle test (*N* = 22^a^)			
Ventilatory threshold (VT)			
• Absolute values (l/min)	1.25 ± 0.36	1.38 ± 0.36	<0.01
• Adjusted for body weight (ml/min/kg)	16.7 ± 4.3	18.5 ± 4.7	<0.01
• VT/VO2 peak (%)	77.6 ± 12.1	78.3 ± 12.3	0.742
Data at exhaustion			
• Maximum workload (Wmax, Watt)	110 ± 52	122 ± 53	<0.01
• Maximum heart rate (bpm)	156 ± 25	161 ± 20	0.16
• Pulmonary ventilation (l/min)	59.6 ± 20.2	68.2 ± 21.0	<0.01
• Tidal volume (l)	1.85 ± 0.48	1.85 ± 0.44	0.91
• Gas exchange ratio	1.15 ± 0.09	1.14 ± 0.08	0.71
Peak oxygen uptake (VO2 peak)			
• Absolute values(l/min)	1.67 ± 0.62	1.82 ± 0.60	<0.01
• Adjusted for body weight (ml/min/kg)	22.1 ± 7.0	24.1 ± 7.1	<0.01
• As % of normal	69.4 ± 17.4	75.9 ± 18.0	<0.01
6-min walk test (*N* = 22)			
• Maximum walking distance (6MWT, m)	492 ± 89	508 ± 97	0.01
Pulmonary function test (*N* = 23)			
• Forced vital capacity (FVC, % of normal)	89.2 ± 12.6	90.0 ± 14.0	0.51

** For the difference before and after training (paired samples t-test)

^a^One patient was excluded because he did not reach the required maximum intensity

#### Core stability

**Fig. 2:**
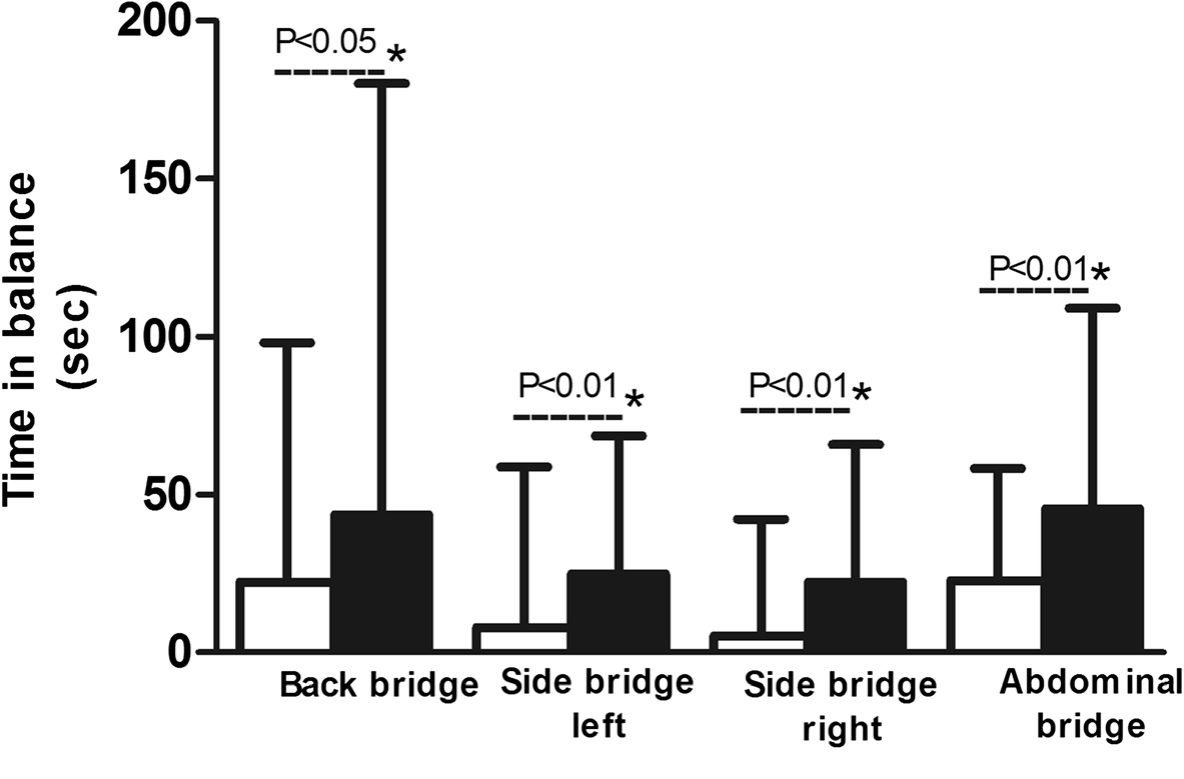
Time patients (*N* = 23) were able to remain in balance for the four different core stability exercises before (white bars) and after training (black bars)

Figure 2 shows the results of the core stability tests. At the start of the program, many patients experienced difficulties in performing the core stability exercises, reporting problems with initiating movement and controlling balance. During the training program, the number of patients who were able to perform the exercises increased for three of the four exercises (from 17 to 21 for the abdominal bridge, 15 to 16 for the left side bridge and 13 to 16 for the right side bridge). The average time they were able to remain in balance improved for all four positions (by 58 % for the back bridge, 229 % for the left and 223 % for the right side bridges, and 86 % for the abdominal bridge; *p* < 0 · 05).

#### Muscle strength

Of the nine muscle groups tested, there were increases in the strength of the hip flexors and shoulder abductors (Table 3).

**Table 3:** Muscle strength measured by hand-held dynamometry (HHD) before and after 12 weeks of training

	Before training	After training	*P*-value*
	Mean ± SD	Mean ± SD	
HHD of individual muscle groups (Newton) (N = 23)			
• Neck extensors	142.1 ± 18.4	140.4 ± 13.8	0.65
• Neck flexors	124.7 ± 40.2	132.1 ± 33.9	0.09
• Shoulder abductors	143.1 ± 29.1	150.7 ± 35.4	0.02
• Elbow flexors	226.4 ± 41.5	230.8 ± 42.3	0.42
• Elbow extensors	180.5 ± 20.1	176.2 ± 25.3	0.27
• Hip flexors	156.4 ± 61.9	180.7 ± 57.7	<0.01
• Hip abductors	159.4 ± 58.3	158.0 ± 68.1	0.75
• Knee extensors	189.2 ± 34.1	193.0 ± 31.8	0.27
• Knee flexors	121.2 ± 57.2	122.6 ± 56.7	0.51

*For the difference before and after training (paired samples t-test

#### Muscle function

Twelve weeks of training significantly reduced the time taken by patients to climb four steps (on average 0.3 sec less, [−0 · 54 to −0 · 04], p = 0.02 Table 4) and to rise from supine to a standing position (1 sec less, [−2.0 to0 · 01], p = 0.05). The QMFT sum score and the time to run 10 meters did not change.

**Table 4:** Muscle function measured by the quantitative motor function test (QMFT) and timed tests before and after 12 weeks of training

	Before training	After training	*P*-value*
	Mean ± SD	Mean ± SD	
QMFT score (N = 22^b^)	51 ± 8	51 ± 9	0.65
Timed Tests (*N* = 22^b^)			
– 10 m running (sec)	4.97 ± 1.50	4.70 ± 1.34	0.16
– Climbing four steps (sec)^a^	2.37 ± 0.80	2.08 ± 0.74	0.02
– Rising from supine to standing position (sec)^a^	5.83 ± 4.25	4.83 ± 2.38	0.05

*For the difference before and after training (paired samples t-test and the Wilcoxon signed-ranks test for paired data^a^)

^b^QMFT score and timed test were not available for one patient

#### Body composition

There were no changes in mineral bone mass (2 · 83 kg ± 0 · 58 before training vs. 2 · 82 kg ± 0 · 57 after training), in lean body mass (42 · 53 kg ± 7 · 99 vs. 43 · 14 kg ± 8 · 28), or in fat mass (30 · 11 kg ± 9.23 vs. 29 · 29 kg ± 9 · 06). Likewise, there were no changes in bone mineral density, overall fat percentage, and android and gynoid fat percentages (results not shown).

### Duration of training effect

After the initial 12 weeks of training group 1 was planned to discontinue training, but 11 of 13 patients chose to continue training with the same or slightly modified intensity. Therefore we were not able to assess how long the training effect maintained after withdrawal.

## Discussion

This study provides clinical evidence that a combination of aerobic, resistance and core stability training can be performed safely in patients with Pompe disease, and helps to improve endurance, core stability and muscle function.

Improved endurance was shown by improved aerobic fitness over 12 weeks of training. In addition to the 11 % increase in maximum workload capacity, peak oxygen uptake and ventilatory threshold improved by 9 % and 10 %, and the 6MWT by 3 %. The increase in peak oxygen uptake was relatively small compared to a number of studies in neuromuscular dystrophies and metabolic myopathies like McArdle disease, showing an increase ranging from 12-47 %. [19–22] In these studies, patients spent more time per week on endurance training, which might explain the larger increase. A second explanation might be the lower baseline VO_2peak_ than in our study.

The baseline values on VO_2peak_ we found in our study were slightly higher than those reported in two Italian studies of Pompe patients (range 15.1 – 26.4 ml/kg/min). [23, 24] These include 8 patients who were assessed prior to treatment (median 20.5 mg/kg/min), and one patient assessed during ERT (15.7 mg/kg/min), while all our patients had been on ERT for at least a year, which might explain the somewhat higher VO_2peak_ values in our cohort. Nevertheless, our 6MWT results also suggest that our cohort had a relatively good endurance capacity before the training (77 % of normal expected). Finally, their ventilatory threshold as a percentage of the peak oxygen uptake (78 %) is relatively high for an untrained population, and comparable to that seen in the three Italian patients that reached the VT (five did not reach the VT in this study, while all our patients reached VT). Our patients thus had relatively good endurance and tolerance capacity before training, which is in agreement with the fact that mildly affected Pompe patients were selected for this study.

So far the 6MWT was used in clinical trials for Pompe disease to assess endurance capacity, but since most patients have walking difficulties it has been questioned whether the 6MWT fully reflects this [25, 26]. Our study indicates that the incremental cycle test offers a good alternative to test endurance capacity, providing insight into patients’ aerobic capacity.

Core stability has not been trained previously in neuromuscular disorders presenting with limb-girdle weakness. One possible reason may lie in the assumption that core stability exercises are not feasible for such patients. Indeed, on starting training, many patients had difficulty performing the exercises. During the program, however, they learned to activate the proper muscle groups and were able to remain in balance for longer. Our results thus indicate that core stability training is feasible and improves time in balance in patients with Pompe disease who are treated with ERT. Feedback from patients during the training also suggests that they perceived their improved core stability to facilitate daily activities. Further studies on how training influences patient reported outcomes such as quality of life are needed.

An increase in muscle strength was observed in two of the nine muscle groups tested: the hip flexors and shoulder abductors. We are not completely certain whether the increased strength of the hip flexors resulted from strength training, core stability exercises, or both. Core stability may support gains in muscle strength by improving proprioception and coordination.

The combined effects of the training program on endurance, core stability, and muscle strength also led to some functional improvement, with patients becoming able to climb four stairs and rise to a standing position faster.

In our study all patients received enzyme replacement therapy. It has been reported that the main incremental effects of enzyme replacement therapy (ERT) are observed during the first year. Therefore only patients who had received ERT more than 1 year were allowed to participate. The study had a staggered design with patients in group 2 starting after a period of no training allowing evaluation of the effect of ERT only, and patients in group 1 scheduled to stop training after 12 weeks with the intention to study how long the effect of training continued. During ERT only patients remained more or less stable indicating that training was the main driver behind the effects. We were not able to assess how long the training effect continued, since all patients in group 1, except two, chose to continue training after 12 weeks. Although patients’ choice interfered with our study design, it also reflects the positive feedback they have given us on the program.

Compliance was high in our study. It is likely that the beneficial effects experienced, the supervision by physiotherapists and weekly telephone consultations contributed to this. We therefore recommend that the program be incorporated into regular supervised physiotherapy sessions.

Few studies have been conducted on exercise and training in Pompe disease. The largest so far was performed before ERT became available [9]. For a mean duration of four years, 26 patients participated in a combined nutrition and endurance exercise therapy program that led to improved muscle function as measured with the Walton score. More recently, a German observational study showed that the effect of ERT on walking distance was most pronounced in five patients who, incidentally, were also subjected to endurance training on a cycle ergometer during ERT infusions [10]. Two other studies, one in mice and one in five adult patients with Pompe disease, however, did not confirm a beneficial effect of endurance training while receiving ERT [27, 28].

Previous exercise studies in Pompe disease mainly focused on endurance training. It has been envisaged that resistance training might lead to muscle damage, thereby aggravating muscle weakness [29–31]. Prior to our study, only Terzis *et al.* [28] combined endurance with resistance training in five patients with Pompe disease receiving ERT. The combined results of these five patients showed that both muscular strength and walking distance improved.

Before starting our study we carefully considered whether we should perform exercise-endurance training only, or a combination of different types of exercises. We chose the latter, because we not only wanted to improve endurance, but also target all affected muscles (resistance exercises), and ameliorate proprioception and the strength of those proximal muscles not targeted by resistance training (core stability exercises). Our decision to use a combined program was also driven by the fact that our patient population was not large enough to run three separate programs.

Although we cannot rule out the possibility that endurance training alone might have had a greater impact on endurance, we observed that the extra exercises had positive effects on core stability, and may also have improved muscle strength and function. Earlier studies in patients with inherited muscular myopathies did not include core stability exercises; our study shows them to be both safe and easy to learn. Patients in our study were mildly affected; those who are more severely affected may need slightly adjusted exercise-training programs but we recommend to include similar components.

## Conclusions

Our study shows that a combination of endurance, strength and core stability training is feasible and can be performed safely in patients with Pompe disease. Such training helps to improve endurance, muscle strength, muscle function and core stability. This training program thus seems to offer added value for Pompe patients to those of ERT.
